# Master Mix Localization Algorithm for Autonomous Systems in Indoor Environments

**DOI:** 10.3390/e28080903

**Published:** 2026-08-12

**Authors:** Zakaryae Ezzouine, Adil Salbi, Mohamed Abouzahir, Ilham Elmourabit, Adil Brouri, Sébastien Roy

**Affiliations:** 1 Research Modeling and Multiphysics Engineering Laboratory (LMIMP), National School of Arts and Crafts, University Moulay Ismail, B.P. 15290 ENSAM, Meknès 50500, Morocco; 2 Electrical Engineering Department, Systems Analysis Information Processing and Integrated Management Laboratory (LASTIMI), High School of Technology, Mohammed V University of Rabat, Avenue Le Prince Héritier, B.P. 227, Salé 11000, Morocco; 3 Engineering Sciences and Biosciences Laboratory (LSIB), Department of Engineering Sciences and Biosciences, Faculty of Sciences and Techniques, Hassan II University of Casablanca, B.P. 9154 Rue Tarik Ibnou Ziad, Casablanca 20360, Morocco; 4 Department of Electrical and Computer Engineering, Sherbrooke University, Sherbrooke, QC J1N 3C6, Canada; roys2121@usherbrooke.ca

**Keywords:** autonomous vehicles (AV), sensor fusion, localization, inertial measurement unit (IMU), LiDAR technology, deep learning, GPS-denied environments, radar, Kalman filtering, signal processing, nonlinear autoregressive with exogenous inputs (NARX)

## Abstract

Reliable navigation in GPS-denied environments remains a critical challenge for autonomous vehicles (AVs), particularly in complex indoor and urban settings. GPS-based localization systems often fail under these conditions, highlighting the need for resilient multimodal solutions. In this article, we present a radar-assisted tracking system that integrates LiDAR and inertial measurements within a sensor-fusion architecture to achieve robust navigation. The principal methodological contribution is a unified tracking and prediction framework that combines Bayesian state estimation with learning-based temporal prediction, enabling accurate tracking while continuously forecasting the slave robot’s short-term future state from mapping observations generated by the master robot, with a typical end-to-end perception-to-action latency of 20–60 ms. The communication and prediction forecasting module operates with an update interval below 35 ms, enabling real-time cooperative robotic operation. Sensor data are fused through a pipeline incorporating Gaussian Mixture Models (GMMs) for post-processing, which helps mitigate the limitations associated with individual sensors during edge processing. Moreover, Kalman filtering is employed to mitigate sensor noise and drift, thereby improving state estimation accuracy through trajectory smoothing. The fused spatiotemporal information is subsequently exploited by a Convolutional Recurrent Neural Network (CRNN) coupled with a Nonlinear Autoregressive model with eXogenous Inputs (NARX) to model the robot’s motion dynamics and provide short-horizon state prediction. Through simulations and real-world indoor experiments conducted in GPS-denied environments, we validate the system’s ability to provide accurate and continuous pose estimation with low localization errors. Experimental results show that the proposed framework achieves root-mean-square errors of 0.12 m, 0.15 m, and 0.28 m along the X, Y, and Z axes, respectively, while maintaining sub-meter maximum position deviations throughout the evaluated trajectories. These results confirm that the proposed framework provides reliable localization and predictive state estimation for cooperative robotic navigation in indoor GPS-denied environments. Future work will investigate outdoor validation and extend the framework to additional data-driven decision-making models for future robotic services.

## 1. Introduction

Target destination estimation and localization are fundamental elements for both intelligent vehicles and autonomous mobile robots, enabling reliable navigation, mapping, and decision making. Traditionally, Global Navigation Satellite Systems (GNSS), including the Global Positioning System (GPS), constitute the backbone of outdoor localization; however, their performance rapidly deteriorates in GPS-denied environments due to satellite signal attenuation, shadowing, multipath propagation, and blockage caused by surrounding structures [1,2]. Consequently, indoor mobile vehicles cannot rely solely on satellite-based positioning and require alternative localization strategies capable of providing continuous and accurate state estimation, which is essential for precise mapping and navigation.

To overcome these limitations, numerous indoor localization techniques have been investigated, including ultra-wideband (UWB), inertial measurement units (IMUs), LiDAR, radar, vision-based systems, and multi-sensor fusion approaches. Recent studies have demonstrated the importance of alternative positioning technologies for GPS-denied environments, where UWB-based solutions and multisource localization strategies have been developed to improve positioning accuracy and robustness [3,4,5]. Moreover, machine-learning-based indoor positioning approaches have gained increasing attention due to their capability to exploit complex relationships between heterogeneous sensor measurements and improve localization performance in challenging environments [6].

Each sensing modality provides complementary advantages but also exhibits inherent limitations. IMUs provide high-rate motion estimation but accumulate drift over time, and LiDAR provides accurate geometric measurements but may be affected by environmental characteristics, while radar demonstrates high robustness under challenging illumination and visibility conditions and provides reliable ranging information in adverse complex environments [7,8].

Therefore, recent research has increasingly focused on combining complementary sensors to exploit their respective strengths while mitigating individual weaknesses through sensor fusion.

Classical Bayesian filtering methods, particularly Kalman-filter-based algorithms, remain among the most widely adopted approaches for estimating position, velocity, and orientation from heterogeneous sensor measurements. These recursive estimation techniques effectively reduce measurement uncertainty, compensate for sensor noise, and improve localization consistency through probabilistic fusion of multiple sensing modalities [9,10,11]. However, filtering performance alone may become insufficient when operating in complex indoor environments characterized by nonlinear sensor behavior and rapidly changing motion patterns. Significant advances in deep learning have motivated the integration of data-driven feature extraction with model-based state estimation. Convolutional Neural Networks (CNNs) are capable of learning discriminative spatial representations from radar and LiDAR measurements, while Long Short-Term Memory (LSTM) networks effectively capture temporal dependencies from sequential sensor data. Hybrid learning architectures combining deep neural networks with classical estimation methods have demonstrated promising improvements in multisensor fusion and localization robustness [8,12,13].

Motivated by these developments, this paper proposes a multi-tracker multisensor fusion framework, referred to as Master Mix (2M), for accurate indoor localization in GPS-denied environments. Rather than relying on a single sensing modality, the proposed framework combines radar, LiDAR, and IMU measurements within a hierarchical estimation architecture that integrates recursive Bayesian filtering with deep sequence learning. The objective is to generate consistent and reliable trajectory estimates while reducing the cumulative effects of sensor uncertainty and inertial drift.

**Figure 1:**
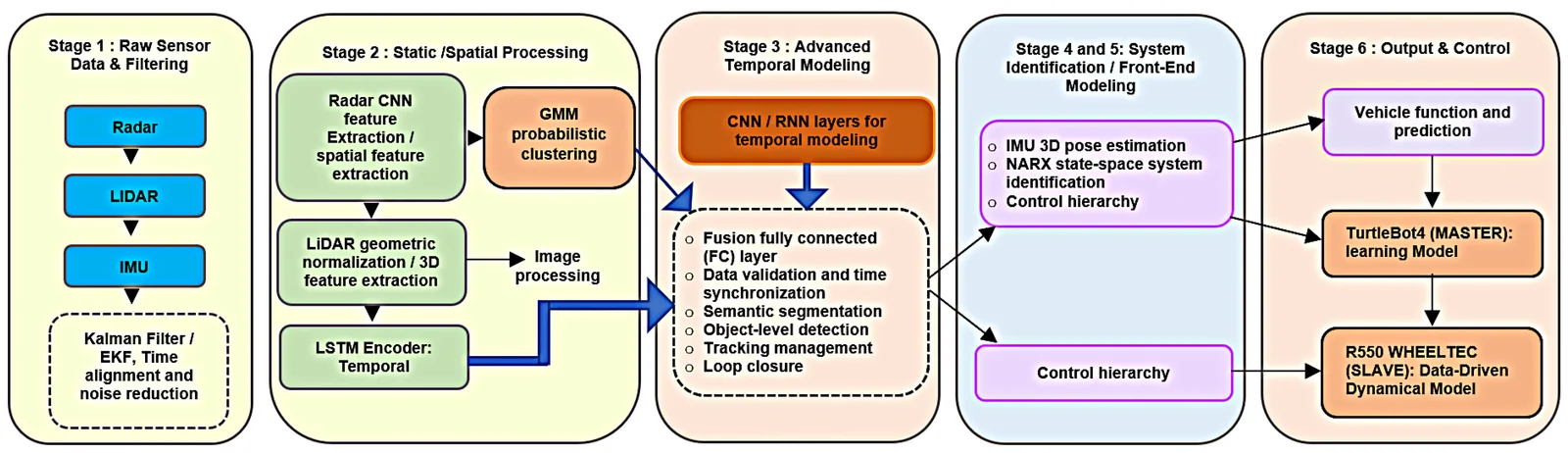
Multi-stage architecture of the proposed multisensor fusion framework for indoor localization.

As illustrated in Figure 1, the overall architecture of the proposed framework consists of six successive processing stages:

- Stage 1: Raw sensor preprocessing and Extended Kalman Filter (EKF)-based state estimation are applied to synchronized radar, LiDAR, and IMU measurements for time alignment, bias compensation, noise reduction, and uncertainty propagation.
- Stage 2: Sensor-specific feature extraction is performed. CNNs process radar range-Doppler-angle representations and LiDAR point-cloud (or BEV) data, while an LSTM models temporal IMU sequences. A Gaussian Mixture Model (GMM) assists with feature clustering and data association.
- Stage 3: A CRNN-based fusion module integrates the extracted spatial and temporal representations to model cross-modal dependencies, enhance temporal consistency and synchronization, and generate robust fused feature representations for localization.
- Stages 4 and 5: The fused states are then processed by a NARX-based system identification model to predict future states, refine trajectory estimation, and produce uncertainty-aware localization outputs.
- Stage 6: The framework also supports cooperative robotic navigation by providing refined pose estimates and accurate localization of the slave robot through exhaustive processing of the information acquired by the primary robot (TurtleBot), thereby closing the perception–estimation–control loop.

By leveraging a multisensor fusion framework combining probabilistic filtering and deep temporal learning, the proposed approach aims to improve localization accuracy, robustness, and trajectory consistency for autonomous mobile robots operating in indoor GPS-denied environments.

## 2. Related Works

Recent advances in Simultaneous Localization and Mapping (SLAM) have significantly accelerated the development of autonomous navigation systems operating in Global Navigation Satellite System (GNSS)-denied environments. By exploiting complementary sensing technologies, modern SLAM frameworks have achieved increasingly reliable localization in complex indoor and outdoor scenarios. Existing approaches can be broadly categorized into three principal sensing paradigms: LiDAR–inertial, visual–inertial, and radar-based SLAM. Each paradigm offers distinct advantages while exhibiting inherent limitations that motivate the development of heterogeneous sensor fusion strategies. LiDAR–inertial systems have emerged as one of the most successful solutions for high-precision localization due to the strong geometric constraints provided by three-dimensional point clouds and the continuous motion estimation offered by inertial sensor measurements. The pioneering LOAM framework introduced a real-time LiDAR odometry and mapping architecture by separating odometry estimation from mapping optimization, thereby achieving an effective balance between computational efficiency and mapping accuracy [14]. Building upon this foundation, LIO-Mapping and LIO-SAM proposed tightly coupled LiDAR–IMU estimation frameworks based on factor graph optimization, inertial preintegration, and loop closure techniques, substantially improving estimation consistency and long-term mapping performance [15,16]. More recently, FAST-LIO and FAST-LIO2 achieved high-frequency state estimation through tightly coupled iterated filtering and direct point-cloud registration, significantly reducing computational complexity while maintaining excellent real-time performance [17,18]. Despite these remarkable achievements, LiDAR–inertial methods remain vulnerable to geometrically degenerate environments such as long corridors, feature-sparse areas, repetitive structures, or partially occluded scenes, where insufficient geometric constraints can degrade localization accuracy. While LiDAR exploits geometric information, visual–inertial SLAM estimates motion by combining image-based feature tracking with inertial measurements. This sensing paradigm benefits from relatively inexpensive hardware and rich environmental information, enabling accurate pose estimation in texture-rich environments. Frameworks such as ORB-SLAM3 integrate visual tracking, inertial optimization, loop closure, and multi-map capabilities within a unified architecture, achieving state-of-the-art localization performance across diverse scenarios [19]. Comprehensive surveys of SLAM research have highlighted the continuous evolution of visual, inertial, and multimodal systems toward increasingly reliable autonomous navigation [20]. Nevertheless, visual–inertial approaches remain highly dependent on environmental appearance and are particularly sensitive to poor illumination, motion blur, dynamic objects, adverse weather conditions, and texture-deficient environments, all of which can substantially reduce localization robustness. Radar-based localization has recently emerged as a promising complementary sensing modality because radar measurements provide reliable range and velocity information under conditions where optical sensors often fail. Unlike cameras and LiDAR, radar performance is largely unaffected by darkness, fog, dust, smoke, or rain, making it particularly attractive for robust autonomous navigation. Recent studies have demonstrated the feasibility of radar-based SLAM using four-dimensional radar data and pose graph optimization, as exemplified by the 4DRadarSLAM framework [21]. Additional research on millimeter-wave and ultra-wideband (UWB) radar has further demonstrated the potential of radar sensing for localization in challenging environments where conventional perception systems experience degraded performance [22,23]. However, radar observations generally exhibit lower spatial resolution, increased measurement noise, clutter, and more ambiguous feature representations than LiDAR data, making accurate feature extraction and data association considerably more challenging when radar is used as a standalone sensing modality. Recognizing the complementary strengths and weaknesses of individual sensors, recent research has increasingly shifted toward heterogeneous multi-sensor fusion for robust localization in GNSS-denied environments. LiDAR provides accurate geometric constraints, radar offers resilience under adverse environmental conditions, and inertial sensors ensure continuous short-term motion estimation during temporary perception degradation. The combination of these complementary sensing characteristics has demonstrated considerable potential for reducing trajectory drift and improving long-term localization reliability. For example, GR-Fusion showed that tightly integrating LiDAR, radar, and IMU measurements significantly enhances localization robustness during long-range navigation by exploiting complementary geometric and motion information [24]. Despite these advances, most existing fusion frameworks rely on handcrafted feature extraction, predefined data association strategies, manually designed measurement models, or fixed probabilistic weighting mechanisms. Such deterministic fusion schemes often limit adaptability when environmental conditions evolve, sensor characteristics vary, or individual sensing modalities become temporarily unreliable [24,25]. In parallel with optimization-based SLAM, learning-enhanced localization has attracted increasing attention by leveraging deep neural networks for feature extraction, place recognition, uncertainty estimation, and adaptive sensor fusion. These approaches have demonstrated encouraging improvements in robustness and perceptual understanding, particularly in highly dynamic or unstructured environments. However, many learning-based methods require extensive training data, often generalize poorly across unseen environments, and may sacrifice the interpretability and probabilistic consistency that characterize classical optimization-based SLAM systems. Consequently, integrating learning techniques within principled probabilistic estimation frameworks remains an active area of research. Infrastructure-assisted indoor positioning systems have also been investigated as an alternative solution for GNSS-denied localization. In particular, Visible Light Positioning (VLP) has demonstrated remarkable positioning accuracy by exploiting dedicated lighting infrastructure and optical signal measurements [26,27]. Nevertheless, these approaches generally depend on specialized infrastructure, controlled illumination conditions, and extensive environmental calibration, limiting their scalability and practical deployment for autonomous mobile robots operating in dynamic and unstructured environments. Collectively, these developments highlight the transition toward heterogeneous sensor fusion as a promising direction for robust SLAM in GNSS-denied environments. Achieving a unified framework that can adaptively integrate complementary sensor information while maintaining robustness under sensor degradation and environmental variability remains an open research challenge.

Under these requirements, robust object detection and localization have become fundamental capabilities for numerous autonomous applications, including mobile robot navigation, autonomous driving, intelligent transportation systems, warehouse automation, and virtual and augmented reality [28,29,30]. Nevertheless, achieving a unified framework that can adaptively exploit complementary sensor information while maintaining robustness under sensor degradation, dynamic environments, and uncertain operating conditions remains an open research challenge. Addressing these limitations is essential for the next generation of intelligent autonomous systems operating in complex real-world environments.

## 3. Theoretical Background

Accurate state estimation is a key component of odometry-based robotic navigation, where accumulated drift, wheel slippage, and measurement uncertainties can significantly degrade localization accuracy. In this work, Kalman Filter (KF) and Extended Kalman Filter (EKF) frameworks are employed to fuse the robot motion model with available measurements for robust state estimation. The KF provides the optimal solution for linear Gaussian systems, whereas the EKF extends this formulation to nonlinear robotic systems through local linearization using Jacobian matrices. The theoretical foundations of both estimators follow the standard formulation reported in [31].

### 3.1. State-Space Formulation

The robot state vector is defined as:

(1) $$x_{k}={\left[\begin{array}{ccccc} x_{k} & y_{k} & \theta_{k} & v_{x,k} & v_{y,k} \end{array}\right]}^{T},$$

where

$x_{k}$, $y_{k}$, and $\theta_{k}$ denote the robot position and orientation, while $v_{x,k}$ and $v_{y,k}$ represent the linear velocity components. The general nonlinear discrete-time system is expressed as:

(2) $$x_{k}=f\left(x_{k-1},u_{k}\right)+w_{k},$$

(3) $$z_{k}=h\left(x_{k}\right)+v_{k},$$

where f(·) and h(·) represent the motion and measurement models, respectively. The process and measurement noises, $w_{k}$ and $v_{k}$, are assumed to be zero-mean Gaussian variables with covariance matrices $Q_{k}$ and $R_{k}$.

### 3.2. KF and EKF State Estimation

For linear systems, the KF formulation is obtained by defining,

(4) $$x_{k}=F_{k}x_{k-1}+B_{k}u_{k}+w_{k},$$

(5) $$z_{k}=H_{k}x_{k}+v_{k}.$$

The recursive prediction and correction stages are given by:

(6) $${\hat{x}}_{k|k-1}=F_{k}{\hat{x}}_{k-1|k-1}+B_{k}u_{k},$$

(7) $$P_{k|k-1}=F_{k}P_{k-1|k-1}F_{k}^{T}+Q_{k},$$

and,

(8) $$K_{k}=P_{k|k-1}H_{k}^{T}{\left(H_{k}P_{k|k-1}H_{k}^{T}+R_{k}\right)}^{-1},$$

(9) $${\hat{x}}_{k|k}={\hat{x}}_{k|k-1}+K_{k}\left(z_{k}-H_{k}{\hat{x}}_{k|k-1}\right),$$

(10) $$P_{k|k}=\left(I-K_{k}H_{k}\right)P_{k|k-1}.$$

For nonlinear robotic motion, the EKF replaces the linear matrices with local approximations obtained from the Jacobians of the nonlinear models:

(11) $$F_{k}={\left.\frac{\partial f(x,u)}{\partial x}\right|}_{{\hat{x}}_{k-1|k-1}},$$

(12) $$H_{k}={\left.\frac{\partial h(x)}{\partial x}\right|}_{{\hat{x}}_{k|k-1}}.$$

Consequently, the EKF prediction step becomes:

(13) $${\hat{x}}_{k|k-1}=f({\hat{x}}_{k-1|k-1},u_{k}),$$

(14) $$P_{k|k-1}=F_{k}P_{k-1|k-1}F_{k}^{T}+Q_{k},$$

while the measurement update follows the same correction structure as the KF using the nonlinear observation function.

The covariance matrices $Q_{k}$ and $R_{k}$ are tuned according to the expected uncertainties of the robot dynamics and measurements. Specifically, $Q_{k}$ accounts for unmodeled effects such as wheel slip and dynamic disturbances, whereas $R_{k}$ represents measurement uncertainties arising from localization sensors, for a step-by-step description of the proposed procedure, refer to Algorithm 1. Proper adjustment of these matrices enables an optimal balance between model prediction and measurement correction, improving the reliability of the estimated robot pose [31].
**Algorithm 1:** Extended Kalman Filter (EKF)
  **Input:** State estimate ${\hat{x}}_{k-1|k-1}$, covariance $P_{k-1|k-1}$, control input $u_{k}$, measurement $z_{k}$

  **Data:** Nonlinear models f(·), h(·), noise covariances $Q_{k}$, $R_{k}$

  **Output:** Filtered state ${\hat{x}}_{k|k}$, covariance $P_{k|k}$

  **Prediction:**

  ${\hat{x}}_{k|k-1}=f\left({\hat{x}}_{k-1|k-1},u_{k}\right)$

  $P_{k|k-1}=F_{k}P_{k-1|k-1}F_{k}^{T}+Q_{k}$

  **Update:**

  $y_{k}=z_{k}-h\left({\hat{x}}_{k|k-1}\right)$

  $S_{k}=H_{k}P_{k|k-1}H_{k}^{T}+R_{k}$

  $K_{k}=P_{k|k-1}H_{k}^{T}S_{k}^{-1}$

  ${\hat{x}}_{k|k}={\hat{x}}_{k|k-1}+K_{k}y_{k}$

  $P_{k|k}=\left(I-K_{k}H_{k}\right)P_{k|k-1}$


### 3.3. Coupled Linear-Predictor-Based Master–Slave Estimation Framework

The proposed estimation framework employs a coupled linear predictor to propagate information between the master and slave estimators. Unlike nonlinear autoregressive models with eXogenous inputs (NARX) [32], which describe nonlinear input-output relationships using past system outputs and external inputs, the proposed formulation relies on linear coupled state-space dynamics. The master estimator provides the latent system evolution, while the slave estimator predicts future states based on the coupled interaction between previous slave states and master information.

This stage constitutes the initial component of the overall pipeline and is subsequently followed by a CNN-based fusion module, which integrates multi-sensor observations to enhance and refine the estimation process. A detailed description of the CNN fusion architecture and its role within the complete framework is provided in Section 4.3, where the overall pipeline is further elaborated. In this formulation, the system dynamics depend both on past estimates and on external observations. The master system provides the underlying latent dynamics, while a slave (data-driven) model performs prediction conditioned on the master evolution.

At each time step, the system first generates a forecast of the next data patch using both its internal state history and the master system evolution. Once the true observation becomes available, the model updates its internal representation, thereby refining future predictions in an autoregressive manner. This mechanism enables the capture of nonlinear temporal dependencies while continuously adapting to newly observed data.

Let $M_{k}$ denote the master system state and $S_{k}$ the slave (data-driven) state. The coupled dynamics are defined as:

(15) $$S_{k+1}^{-}=A_{s}S_{k}+B_{s}M_{k},$$

(16) $$M_{k+1}=A_{m}M_{k}+w_{k},$$

where $A_{s}$ governs the internal evolution of the slave model, $B_{s}$ encodes the influence of the master system, and $w_{k}$ represents process noise.

These dynamics can be written in compact form using the augmented coupling matrix:

(17) $$C=\left[\begin{array}{cc} A_{m} & 0\\ B_{s} & A_{s} \end{array}\right],\;\left[\begin{array}{c} M_{k+1}\\ S_{k+1} \end{array}\right]=C\left[\begin{array}{c} M_{k}\\ S_{k} \end{array}\right]+\left[\begin{array}{c} w_{k}\\ 0 \end{array}\right].$$

The estimation follows a prediction–correction (NARX-like) cycle over *T* time steps. At each iteration, the slave model produces a forecasted patch, which is then corrected using the newly observed measurement.

## 4. Materials and Methods

### 4.1. Components and Instrumentation

**Figure 2:**
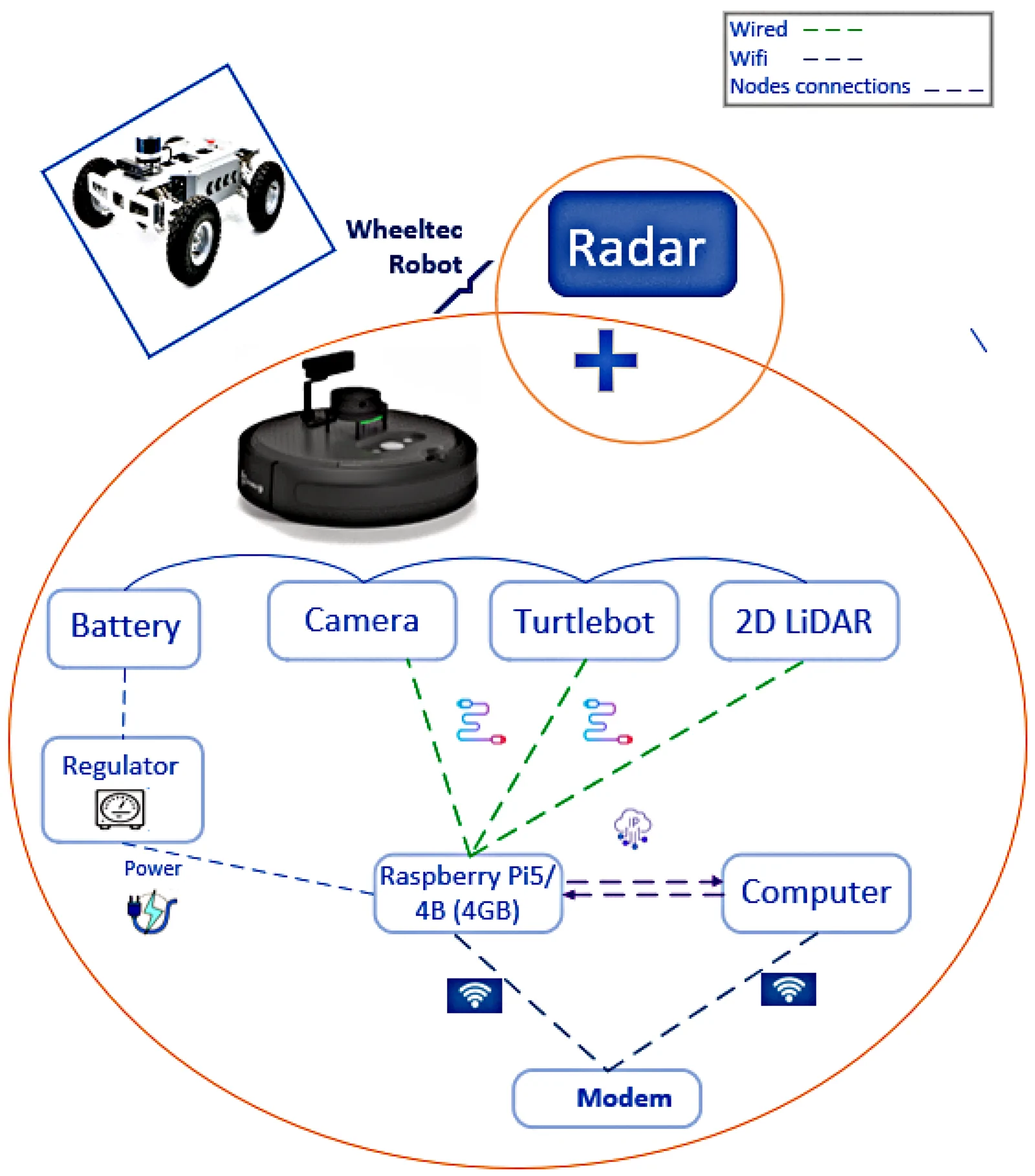
System architecture showing the main components and their interconnections. Blue dashed lines represent Wi-Fi data links, while violet dashed arrows indicate 4G IP-based communication between the Raspberry Pi 5 and the computer. Green lines denote USB serial/data links connecting the TurtleBot camera and the 2D LiDAR. The battery provides the primary power source, which is regulated by a voltage regulator and subsequently distributed to all connected system components and peripherals through the central power interface.

This section provides an overview of the physical components and platforms used in the experimental setup, as illustrated in Figure 2.

#### 4.1.1. TurtleBot Robot

The TurtleBot 4 is a modern mobile robotic platform designed for research and education. Developed by Clearpath Robotics in collaboration with iRobot, it integrates the iRobot^®^ Create 3 base with additional sensing, compute, and communication modules, and is fully compatible with ROS 2. The platform includes built-in sensors such as an IMU, cliff and bump sensors, and wheel encoders. Its modular design facilitates the integration of external sensors and computing units, enabling advanced autonomous navigation and perception tasks. The platform was sourced from Clearpath Robotics, Kitchener, Ontario, Canada (https://clearpathrobotics.com/).

#### 4.1.2. LiDAR

A 2D LiDAR sensor is employed to obtain geometric information about the robot’s surrounding environment. The sensor provides a full 360° scan composed of 1000+ distance readings per revolution, with a measurement range typically extending from 0.12 m to 12 m, depending on the specific LiDAR model used on the TurtleBot 4 platform. The resulting scan is delivered as an array of range values with an angular resolution on the order of 0.25–0.33°, enabling accurate mapping and obstacle detection. The LiDAR system is from Hesai Technology, Palo Alto, CA, USA (https://www.hesaitech.com/).

#### 4.1.3. Onboard Compute Module

The onboard compute module is a Raspberry Pi 5, manufactured by Raspberry Pi Ltd. in the United Kingdom (Wales) (https://www.raspberrypi.com/), which acts as the primary processing unit for the TurtleBot 4. With a quad-core ARM Cortex-A72 processor and up to 8 GB of RAM, the onboard computing module supports ROS 2 operations and sensor fusion tasks, providing sufficient computational capabilities for mid-level autonomous navigation and simultaneous localization and mapping (SLAM) algorithms. The computing setup is complemented by Mac and Lenovo systems for additional processing, data analysis, and experiment management.

#### 4.1.4. WHEELTEC R550

Manufactured by WHEELTEC (https://wheeltec.net/) in Dongguan, China, the R550 is a modular mobile robot platform supporting multiple chassis configura- tions—including Ackermann, 4WD, Mecanum, and tracked designs—enabling versatile mobility for research and educational applications. Equipped with a Raspberry Pi 5 processor and a modular sensor suite—including LiDAR, depth/RGB cameras, and environmental sensors—it supports autonomous navigation, SLAM, and perception experiments, making it an adaptable and robust platform for both indoor and outdoor robotic research.

**Figure 3:**
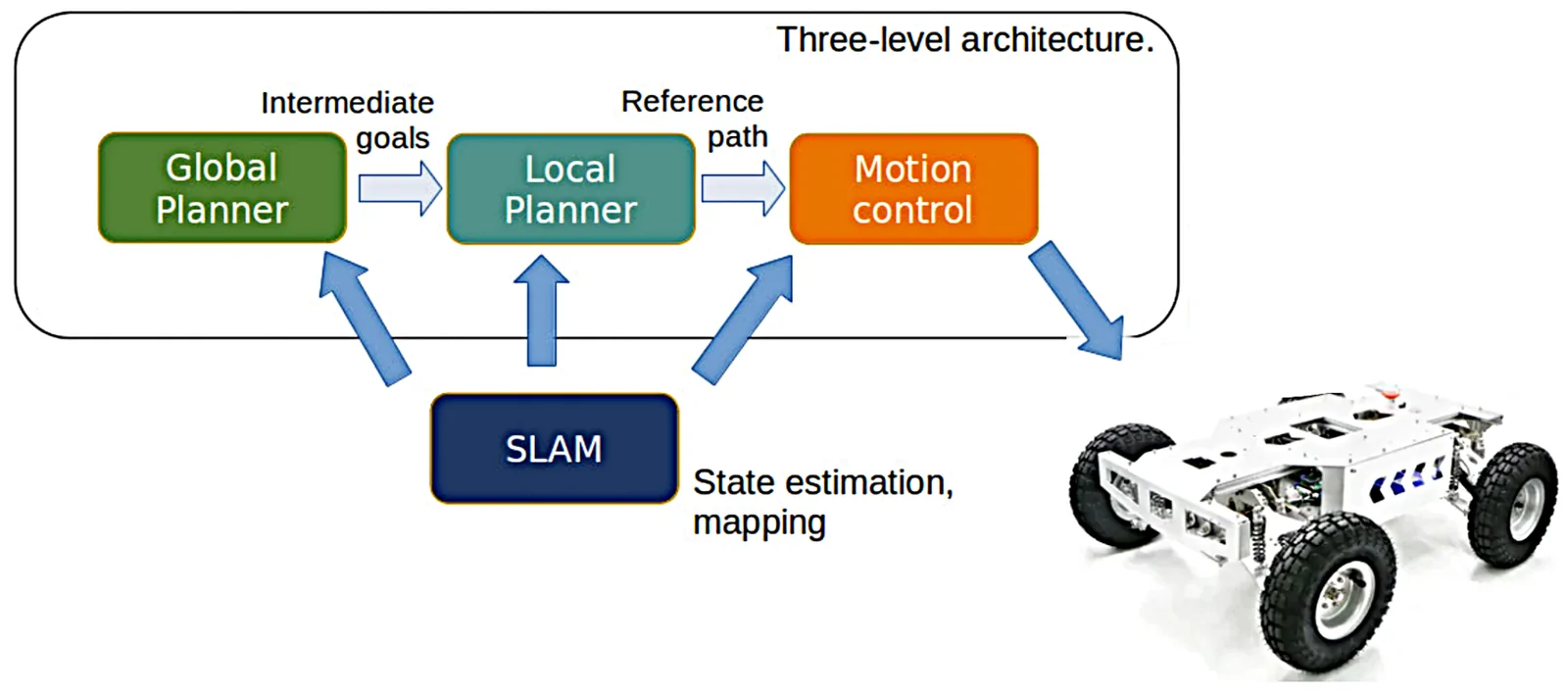
Three-layer decision-making framework.

We evaluated the proposed method indoors using the R550, as shown in Figure 3. The system was initially validated with the TurtleBot 4, followed by experiments on the R550, which provided a modular chassis and an advanced sensor suite for flexible indoor measurements. This setup allowed for precise data collection and a wide range of environmental interactions critical for comprehensive assessments.

The sensor suite included the following components:

- Hesai Pandar40 LiDAR: A 40-channel 3D LiDAR providing full 360° horizontal and approximately 40° vertical coverage for detailed point-cloud data.
- Delphi ESR (Electronically Scanning Radar): A 77 GHz automotive millimetre-wave radar sensor designed for long-range object detection and dynamic tracking in ADAS applications. It employs frequency-modulated continuous-wave (FMCW) technology with electronically scanned antennas to estimate target range, relative velocity, and angular position. The sensor can detect and track up to 64 targets simultaneously, providing range, angle, and velocity information with a detection range of approximately 200–300 m, range resolution of 0.1–0.5 m, and angular accuracy of 1–2°. The compact radar module (approximately 20–40 cm^2^ mounting footprint) was installed at the front centre of the R550 vehicle to provide an unobstructed forward field of view and accurate target detection. The equipment is proposed by AutonomouStuff, Morton, IL, USA (https://autonomoustuff.com/).
- GNSS/IMU (XW-GI7660): Provides position, velocity, and orientation data to support sensor fusion and pose estimation.

Data collection was conducted in indoor environments to emulate GPS-based positioning using numeric data from both the hardware and target systems; no outdoor experiments were performed, as these are planned for future work. For temporal synchronization, hardware timestamps were employed for LiDAR and GNSS/IMU measurements, while ROS common clocks ensured alignment of all data streams at the software level, enabling consistent fusion and logging. A summary of the sensor specifications is provided in Table 1.

### 4.2. Methods

#### 4.2.1. Radar Data Acquisition and Processing

The proposed framework employs an *ESR* 77 GHz automotive radar as an additional sensing modality to complement LiDAR and IMU measurements. Unlike LiDAR, which provides dense geometric point clouds, the radar produces sparse detections containing target range, radial velocity (Doppler), azimuth angle, and signal intensity.

Each radar frame is represented as:

(18) $$R_{t}={\left\{\left(r_{i},\;\theta_{i},\;v_{i},\;\gamma_{i}\right)\right\}}_{i=1}^{N},$$

where

- $r_{i}$ denotes the target range;
- $\theta_{i}$ denotes the azimuth angle;
- $v_{i}$ denotes the Doppler (radial) velocity;
- $\gamma_{i}$ denotes the radar signal intensity.

Prior to sensor fusion, radar detections undergo the following preprocessing steps:

- Clutter removal;
- Outlier rejection;
- Coordinate transformation;
- Timestamp synchronization.

These preprocessing operations are performed using the ROS 2 TF framework to ensure spatial and temporal consistency with the LiDAR and IMU measurements.

Unlike LiDAR, which produces dense three-dimensional point clouds, radar measurements are considerably sparser but provide valuable dynamic information through Doppler velocity estimation. Consequently, radar contributes complementary motion cues that improve localization robustness under varying illumination and adverse environmental conditions. The preprocessed radar observations are then forwarded to the radar branch of the proposed Convolutional Recurrent Neural Network (CRNN), where discriminative feature vectors are extracted before multimodal fusion with the LiDAR and IMU features.

#### 4.2.2. Multi-Tracker Fusion Framework Strategy

**Figure 4:**
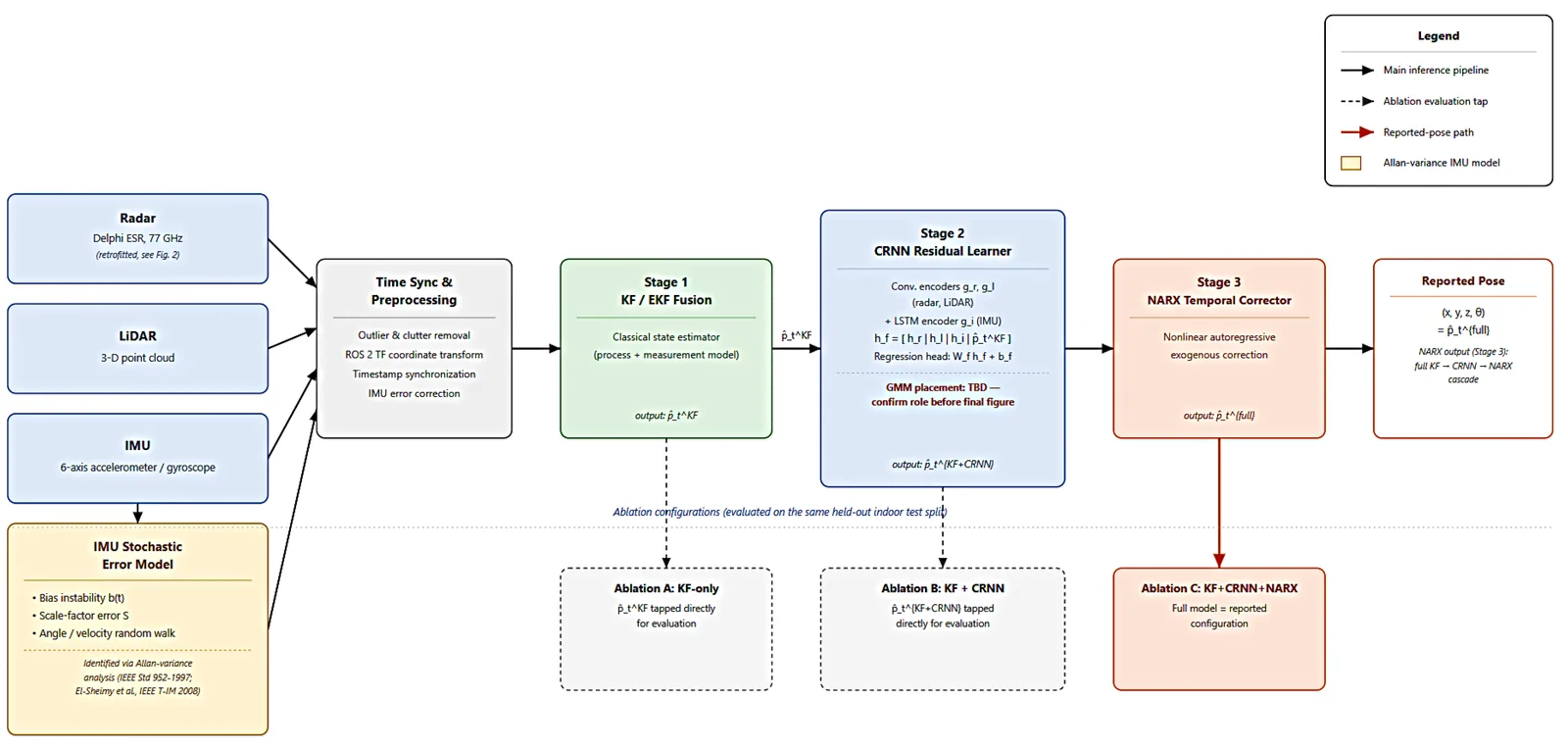
Proposed CRNN multi-sensor fusion architecture [33].

Decision Fusion Level (DFL) is a multimodal sensor fusion strategy in which the outputs or decisions from multiple sensing modalities are combined to produce a final localization estimate. The framework integrates radar, LiDAR, and inertial measurements using a deep learning model, as illustrated in Figure 4.

Unlike traditional Bayesian fusion (e.g., EKF or UKF-based approaches [34,35]), Our method constructs a learned latent state from heterogeneous inputs, enabling the network to infer sensor correspondences, handle nonlinearities, and improve robustness in degraded environments. Each sensor contributes unique, complementary information:

- Radar enables accurate target detection and ranging under varying illumination conditions.
- LiDAR offers dense 3D structure for fine-grained spatial mapping.
- IMU captures high-rate motion dynamics but suffers from drift.

These sensors differ in dimensionality, sampling rate, and noise characteristics, requiring a fusion strategy capable of learning cross-sensor associations and joint representations.

Let $R_{t}$, $L_{t}$, and $I_{1:t}$ denote the radar, LiDAR, and IMU data streams. The goal is to learn a function:

(19) $$f_{\theta }:\left(R_{t},L_{t},I_{1:t}\right)\to {\hat{p}}_{t}$$

where ${\hat{p}}_{t}=\left(x_{t},y_{t},z_{t}\right)$ represents a 3D pose, or ${\hat{T}}_{t}=\left(x_{t},y_{t},z_{t},\phi_{t},\theta_{t},\psi_{t}\right)$ for a full pose estimate.

Feature extraction is expressed as:

(20) $$h_{r}=g_{r}\left(R_{t}\right),\;h_{l}=g_{l}\left(L_{t}\right),\;h_{i}=g_{i}\left(I_{1:t}\right),$$

where $g_{r}\left(\cdot \right)$ and $g_{l}\left(\cdot \right)$ denote convolutional feature encoders for radar and LiDAR data, respectively, while $g_{i}\left(\cdot \right)$ represents the recurrent LSTM encoder that models temporal dependencies within the inertial measurements. The extracted latent representations are concatenated into a unified multimodal feature vector,

(21) $$h_{f}=\left[h_{r}\;\|\;h_{l}\;\|\;h_{i}\;\|\;{\hat{x}}_{t}^{KF}\right],$$

where ‖ denotes vector concatenation, r denotes radar measurements, l denotes LiDAR measurements, i denotes IMU measurements, and ${\hat{x}}_{t}^{KF}$ is the estimated vehicle pose at time *t*.

### 4.3. Cross-Modal Calibration and Robustness

The CRNN fusion architecture learns to calibrate radar, LiDAR, and IMU data, improving localization accuracy by leveraging their complementary characteristics.

Radar provides coarse long-range features, LiDAR delivers high-precision spatial information, and the IMU supplies high-frequency motion measurements. These complementary features are concatenated into a unified latent representation, and the fusion layer learns a nonlinear mapping to capture cross-modal correlations while accounting for sensor noise, environmental variability, and vehicle dynamics. The resulting latent representation is modeled using a Gaussian Mixture Model (GMM) to better characterize its multimodal distribution. The fused feature vector is then denoted as indicated in Equation (21). The probability density of the fused representation is approximated as:

(22) $$p\left(h_{f}\right)=\sum_{k=1}^{K}\pi_{k}\;N\left(h_{f}|\mu_{k},\Sigma_{k}\right),$$

where

- *K* denotes the number of Gaussian components;
- $\pi_{k}$ is the mixture weight;
- $\mu_{k}$ represents the mean vector;
- $\Sigma_{k}$ denotes the covariance matrix of the $k^{\mathrm{th}}$ Gaussian component.

Rather than replacing the classical filtering framework, the proposed CRNN and NARX stages operate as trainable residual correction modules applied on top of the first-stage KF/EKF estimate, ${\hat{p}}_{t}^{\mathrm{KF}}$. The GMM does not directly generate the final localization output; instead, it models the probability distribution of the learned multimodal latent feature space. This probabilistic representation captures multiple plausible vehicle states caused by noisy radar observations, LiDAR degradation, and inertial uncertainty, enabling the subsequent temporal prediction network to refine the remaining position and heading residuals. Therefore, the GMM serves as an intermediate probabilistic refinement layer between the CRNN feature extractor and the NARX-based prediction module, improving robustness without requiring manual re-tuning of filter parameters across different environments.

### 4.4. IMU Error Modeling and Drift Mitigation

Inertial Measurement Units (IMUs) are affected by several systematic and stochastic error sources that accumulate over time, leading to localization drift. Although IMUs provide highly accurate short-term motion estimates, uncompensated errors progressively degrade localization accuracy during long-term navigation.

The inertial measurement model adopted in this work considers the dominant error sources encountered in practical MEMS-based inertial sensors,

(23) y=s⊙x+b+n,

where x denotes the true inertial signal, s represents the scale-factor error, b is the slowly varying sensor bias, and n corresponds to zero-mean random measurement noise.

The principal IMU error sources considered in the proposed localization framework include:

- Bias instability, producing slowly varying offsets in accelerometer and gyroscope measurements.
- Scale-factor error, caused by manufacturing tolerances and calibration inaccuracies.
- Random walk, resulting from stochastic measurement noise accumulated through temporal integration.

The cumulative drift generated by these error sources can be expressed as:

(24) $$\epsilon \left(t\right)=\epsilon_{0}+\int_{0}^{t}n\left(\tau \right)\;d\tau ,$$

where ϵ(t) denotes the accumulated drift at time *t*, $\epsilon_{0}$ is the initial drift, and n(t) represents the time-varying stochastic IMU noise process.

To mitigate inertial drift, the proposed framework combines IMU measurements with complementary radar and LiDAR observations within the three-stage localization architecture. The KF/EKF edge provides short-term state estimation and suppresses measurement noise, while the CRNN learns nonlinear relationships among synchronized radar, LiDAR, and IMU measurements. Finally, the NARX predictor exploits temporal dependencies to compensate for long-term drift that cannot be corrected by conventional filtering alone. Consequently, accumulated inertial errors are significantly reduced without requiring repeated manual tuning of filter parameters.

Furthermore, the multimodal fusion strategy improves robustness under varying environmental conditions by adaptively exploiting the complementary characteristics of each sensing modality. Radar measurements remain reliable under poor visibility and partial occlusions, LiDAR provides highly accurate geometric information in feature-rich environments, and the IMU ensures continuous motion estimation during rapid vehicle dynamics or temporary degradation of exteroceptive sensors. This complementary fusion enables stable and accurate localization in challenging GNSS-denied environments.

### 4.5. Deep Latent-State Fusion

The CRNN architecture generates three latent embeddings:

(25) $$h_{r}=g_{r}\left(R_{t}\right),\;h_{l}=g_{l}\left(L_{t}\right),\;h_{i}=g_{i}\left(I_{1:t}\right)$$

where $R_{t}$ is the radar input at time *t*, $L_{t}$ is the LiDAR input at time *t*, and $I_{1:t}$ represents the IMU sequence from time 1 to *t*. The functions $g_{r}\left(\cdot \right)$, $g_{l}\left(\cdot \right)$, and $g_{i}\left(\cdot \right)$ are learned feature encoders for each modality.

These embeddings are fused into a single representation:

(26) $$h_{f}=\left[h_{r}\;\|\;h_{l}\;\|\;h_{i}\right]$$

where ‖ denotes vector concatenation along the feature dimension.

This fused representation is then passed through a regression network to predict the vehicle pose:

(27) $${\hat{p}}_{t}=W_{f}h_{f}+b_{f}$$

where $W_{f}$ is the learned weight matrix of the regression head and $b_{f}$ is the bias term.

Vehicle localization is inherently sequential since the current vehicle state depends strongly on previous observations and motion history. To exploit these temporal dependencies, the probabilistic feature representation generated by the GMM is supplied to a Nonlinear AutoRegressive Network with eXogenous inputs (NARX).

Unlike conventional recurrent predictors, the NARX model explicitly considers both previous localization estimates and the current multimodal feature vector. The predicted vehicle pose is therefore expressed as:

(28) $${\hat{x}}_{t}=F\left({\hat{x}}_{t-1},\ldots ,{\hat{x}}_{t-d},z_{t},\ldots ,z_{t-d}\right),$$

where

- F(·) denotes the nonlinear NARX mapping;
- *d* represents the temporal delay order;
- $z_{t}$ denotes the probabilistic feature vector produced by the GMM.

The final localization output therefore corresponds to

(29) $${\hat{x}}_{t}={\left[\hat{x},\hat{y},\hat{z},\hat{\phi },\hat{\theta },\hat{\psi }\right]}^{T},$$

which represents the estimated six-degree-of-freedom vehicle pose.

Compared with the initial KF estimate, the NARX predictor learns nonlinear residual corrections from previous localization history, thereby reducing long-term drift while preserving temporal consistency.

### 4.6. Sensor Feature Extraction

Radar and LiDAR data are processed using convolutional layers to extract spatial features:

(30) $$F_{r}=\sigma \left(W_{r}*R_{t}+b_{r}\right),\;F_{l}=\sigma \left(W_{l}*L_{t}+b_{l}\right)$$

where $F_{r}$ and $F_{l}$ are the extracted radar and LiDAR feature maps, $W_{r}$ and $W_{l}$ are convolution kernels, $b_{r}$ and $b_{l}$ are biases, ∗ denotes convolution, and σ(·) is a nonlinear activation function (e.g., ReLU).

IMU data is processed using an LSTM to capture motion continuity and mitigate drift:

(31) $$h_{i}=\mathrm{LSTM}\left(I_{1:t}\right)$$

where $h_{i}$ is the IMU temporal embedding and $I_{1:t}$ is the sequential IMU input.

### 4.7. Final Pose Estimation

The fused latent vector is passed through a regression head to predict the vehicle pose:

(32) $${\hat{p}}_{t}=W_{f}h_{f}+b_{f}$$

where ${\hat{p}}_{t}$ is the predicted pose, $h_{f}$ is the fused feature vector, $W_{f}$ is the regression weight matrix, and $b_{f}$ is the bias term.

The training objective minimizes pose estimation error:

(33) $$L_{\mathrm{MSE}}={∥{\hat{p}}_{t}-p_{t}^{\mathrm{GT}}∥}_{2}^{2}$$

where $p_{t}^{\mathrm{GT}}$ is the ground-truth pose at time *t* and ${∥\cdot ∥}_{2}^{2}$ denotes the squared Euclidean norm.

#### 4.7.1. Deep Learning Fusion Architecture

A Convolutional Recurrent Neural Network (CRNN) is used to combine multimodal sensor data by jointly modeling spatial features and temporal dynamics across all input streams. Radar and LiDAR are processed by two CNN encoders, while the IMU stream is handled by an LSTM encoder. Early-stage feature extraction is modality-specific, whereas high-level fusion is achieved by concatenating latent embeddings.

#### 4.7.2. Model Training

The proposed localization framework is trained end-to-end using synchronized radar–LiDAR–IMU sequences acquired from the experimental platform. The collected dataset consists of **N** complete trajectories recorded in the indoor experimental environment. To preserve temporal consistency and prevent data leakage, the trajectories are divided into training, validation, and testing subsets using a 70%/15%/15% split, corresponding to $N_{train}$, $N_{val}$, and $N_{test}$ trajectories, respectively. Each complete trajectory is assigned exclusively to one subset to ensure an unbiased evaluation of the proposed localization framework.

Before multimodal fusion, radar measurements are preprocessed to remove spurious detections and clutter, then synchronized with the corresponding LiDAR scans and IMU measurements. The processed sensor data are subsequently passed through the unified localization pipeline, consisting of the Kalman Filter (KF/EKF), CRNN-based feature fusion module, Gaussian Mixture Model (GMM) representation, and NARX prediction module.

Training is performed using the Adam optimizer [36] with an initial learning rate of $10^{-3}$. The optimization minimizes the mean squared error between the predicted vehicle pose and the corresponding ground-truth localization measurements.

(34) $$L=\frac{1}{N}\sum_{i=1}^{N}{∥{\hat{x}}_{i}-x_{i}^{GT}∥}_{2}^{2},$$

where $x_{i}^{GT}$ denotes the ground-truth pose and ${\hat{x}}_{i}$ represents the predicted localization output.

The validation subset is used exclusively for hyperparameter tuning and early stopping, whereas the testing subset is reserved for the final performance evaluation. The complete localization framework combines model-based state estimation with deep feature learning to exploit both the physical consistency provided by classical filtering and the nonlinear representation capability of deep neural networks.

The proposed CRNN architecture is illustrated in Figure 4. The network receives the initial KF/EKF pose estimate together with synchronized radar, LiDAR, and IMU observations. Separate convolutional feature extractors are employed for radar and LiDAR measurements, while IMU observations are processed through an LSTM branch to capture temporal dependencies. The extracted feature representations are concatenated and projected into a common latent feature space. A Gaussian Mixture Model (GMM) is then used to represent the probabilistic distribution of the fused latent representation, and the resulting state representation is provided to the NARX predictor to estimate the final vehicle pose while considering temporal correlations in the localization sequence.

The network is trained for 100 epochs using a batch size of 16. Early stopping based on the validation loss is applied to prevent overfitting, and a learning-rate scheduler is used to improve convergence during training. The complete framework was developed using PyTorch 2.9.0 under Python 3.13 for computational processing and model development. MATLAB R2025a was used alongside the Python-based implementation for the sensor-fusion pipeline, numerical processing, and the generation of the corresponding experimental visualizations. The framework was trained on a workstation equipped with an NVIDIA RTX 4090 GPU, an Intel Core i9 processor, and 64 GB of RAM. The average training duration is approximately 6 h, while the average inference latency is approximately 8.2 ms per frame, enabling real-time localization.

The adopted training configuration remains fixed for all experiments to ensure a fair comparison between the different localization approaches. The main parameters include the Adam optimizer, an initial learning rate of $10^{-3}$, the CNN-based radar and LiDAR feature extractors, the LSTM-based IMU encoder, the GMM latent representation module, and the NARX temporal prediction network.

To quantify the contribution of each processing stage, an ablation study is conducted by progressively enabling the proposed modules. Specifically, localization performance is evaluated using (i) the Stage 1 KF/EKF baseline, (ii) the KF/EKF combined with the CRNN feature fusion network, and (iii) the complete platefrom framework. The corresponding results are reported in Section 5. In addition, radar-only localization and radar-LiDAR-IMU fusion results are compared to demonstrate the contribution of radar sensing to the overall localization accuracy.

### 4.8. Performance Metrics

The performance of the proposed CRNN–NARX prediction model was evaluated using quantitative trajectory prediction metrics. For each prediction horizon, the predicted robot position was compared with the corresponding ground-truth trajectory obtained from the reference localization system. The Root Mean Square Error (RMSE) and Mean Absolute Error (MAE) were adopted to evaluate prediction accuracy and quantify the increase in uncertainty over extended forecasting horizons.

The RMSE metric measures the overall deviation between the predicted and reference positions and is defined as:

(35) $$RMSE=\sqrt{\frac{1}{N}\sum_{i=1}^{N}{\left(y_{i}-{\hat{y}}_{i}\right)}^{2}}$$

where *N* represents the number of prediction samples, $y_{i}$ denotes the ground-truth position, and ${\hat{y}}_{i}$ represents the predicted position at the corresponding prediction horizon.

The MAE metric is used to evaluate the average absolute prediction error and is expressed as:

(36) $$MAE=\frac{1}{N}\sum_{i=1}^{N}\left|y_{i}-{\hat{y}}_{i}\right|$$

Both metrics were calculated independently for each prediction horizon, including 0.2 s, 0.5 s, 1 s, 2 s, and 3 s. This evaluation provides a quantitative analysis of the prediction accuracy degradation associated with uncertainty accumulation during extended state forecasting.

### 4.9. Ablation Study

To evaluate the contribution of each component of the proposed localization framework, an ablation study is conducted by progressively integrating the different processing stages. The objective of this analysis is to quantify the individual contribution of the Stage 1 KF/EKF estimation, the CRNN-based multimodal feature fusion module, the GMM probabilistic representation, and the NARX temporal prediction network.

Three configurations are considered in the evaluation:

- **Model A** (KF/EKF baseline):The initial vehicle pose is estimated using only the Stage 1 Kalman Filter (KF/EKF), without any deep-learning-based refinement. This configuration represents the conventional model-based localization approach.
- **Model B** (KF/EKF + CRNN): The KF/EKF estimation is enhanced using the CRNN multimodal fusion network. In this configuration, radar, LiDAR, and IMU measurements are processed through modality-specific feature extraction branches, and the extracted representations are combined to refine the initial pose estimate.
- **Model C** (KF/EKF + CRNN + GMM + NARX): The complete proposed framework, where the CRNN extracted features are modeled using a Gaussian Mixture Model (GMM) to represent uncertainty in the latent feature space. The resulting probabilistic representation is then processed by the NARX predictor to exploit temporal dependencies and provide the final refined localization output.

All experiments are performed using the same testing trajectories and identical environmental conditions to ensure a fair comparison between the different configurations. The localization performance is evaluated using the Root Mean Square Error (RMSE) and Mean Absolute Error (MAE) metrics defined previously.

The ablation results demonstrate the progressive improvement obtained by introducing each proposed component. The KF/EKF baseline provides an initial physically consistent estimation; however, its performance is affected by sensor noise accumulation and nonlinear environmental variations. The integration of the CRNN module improves localization accuracy by learning nonlinear relationships between heterogeneous radar, LiDAR, and IMU measurements. The additional integration of the GMM and NARX modules further reduces the localization error by modelling uncertainty in the fused latent representation and exploiting temporal correlations within the trajectory sequence.

Table 2 compares the TPF configurations in terms of RMSE, MAE, and localization improvement. Among the evaluated configurations, Model C achieves the lowest localization error, with an RMSE reduction from 0.42 m to 0.13 m compared with the TPF baseline, corresponding to an improvement of approximately 69%. This result demonstrates the effectiveness of combining classical state estimation with deep multimodal representation learning.

The results also highlight the individual contribution of the CRNN feature fusion module. By integrating radar, LiDAR, and IMU observations into a shared latent representation, the CRNN reduces the RMSE by approximately 42.9% compared with the KF/EKF-only configuration. Furthermore, the additional improvement obtained after introducing the GMM and NARX modules demonstrates the importance of probabilistic modelling and temporal prediction for long-term localization consistency.

Furthermore, the contribution of radar sensing is evaluated by comparing radar-only localization with the complete radar–LiDAR–IMU fusion framework. The fusion approach provides improved robustness compared with single-sensor localization, particularly in indoor environments where LiDAR observations may be degraded by occlusions, limited geometric features, or dynamic obstacles. These results confirm that radar provides complementary motion and range information that enhances the overall reliability of the proposed cooperative localization framework. Further analysis of the radar contribution is provided in the following section.

## 5. Results and Discussion

The 2M fusion system integrates radar, IMU, and LiDAR data to achieve robust positioning for autonomous vehicles, particularly in environments where GNSS signals are weak or unavailable. Experimental results from indoor campus environments demonstrate that the 2M system provides accurate trajectory estimates and reliable navigation even under challenging conditions such as sensor dropouts and occlusions. Building upon this multimodal localization capability, the proposed cooperative framework further integrates prediction and communication mechanisms to enhance robotic navigation performance.

The cooperative localization and predictive framework was evaluated through both simulation and real-world indoor experiments conducted in GNSS-denied environments. Within the proposed architecture, the master robot acquires multimodal sensor observations to generate localization information, which is subsequently processed and transmitted to the slave robot through a low-latency communication framework. The complete sensing, communication, and forecasting pipeline operates with an end-to-end latency below 35 ms, including sensor acquisition, edge processing, data transmission, and predictive state estimation. This low-latency operation enables continuous localization updates and efficient information exchange between cooperating robots during navigation.

To evaluate the forecasting capability of the proposed framework, the trained CRNN–NARX model was tested over multiple future-state horizons of 0.2 s, 0.5 s, 1 s, 2 s, and 3 s. For each evaluation interval, the estimated future trajectory of the slave robot was compared with the corresponding ground-truth trajectory obtained from the reference localization system. Prediction performance was quantified using the Root Mean Square Error (RMSE) and Mean Absolute Error (MAE), providing a quantitative assessment of prediction accuracy degradation with increasing forecasting horizons, as reported in Table 3.

**Figure 5:**
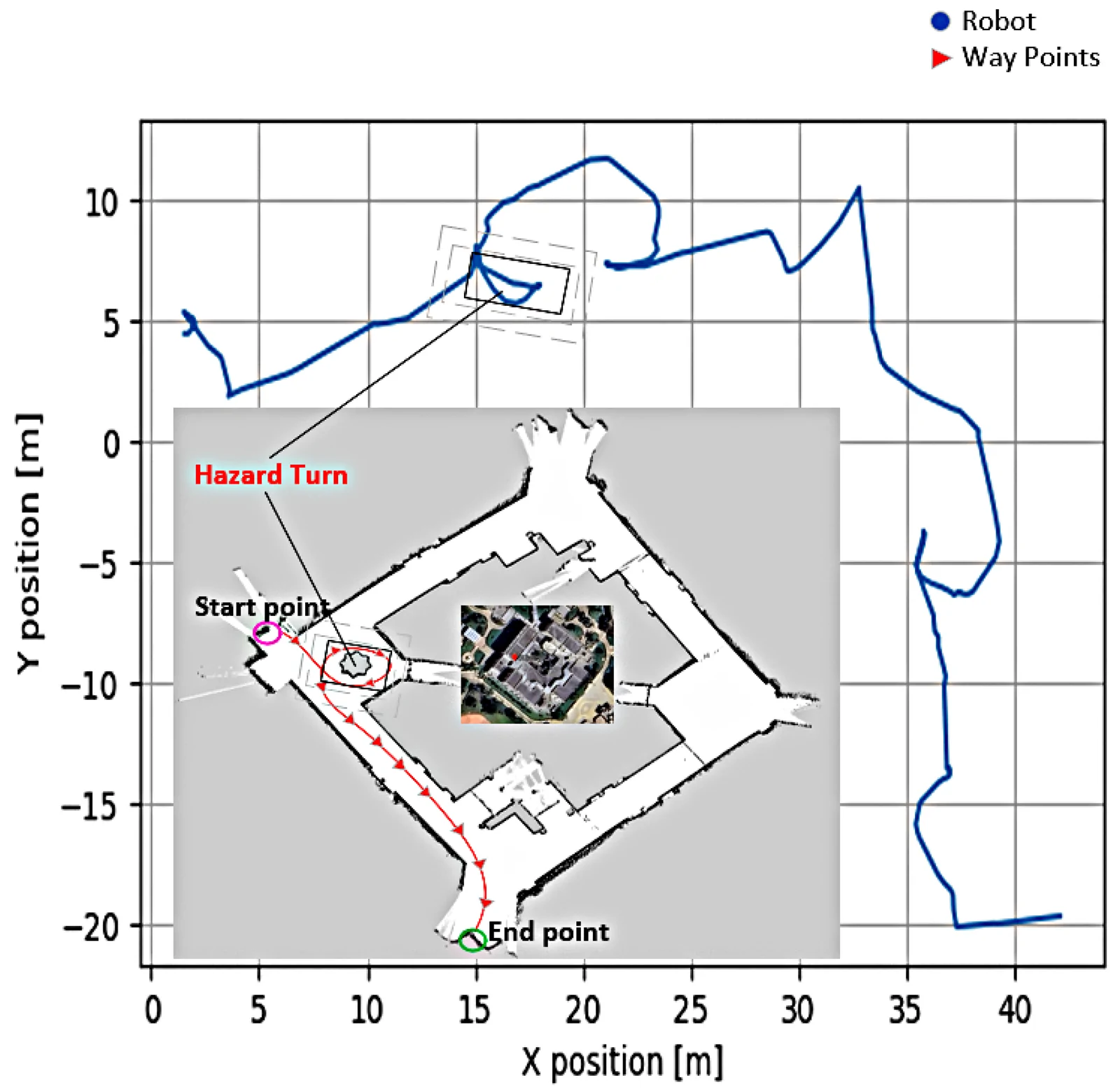
Real Robot Trajectory Map—Indoor Environment (image taken from the campus dataset).

As shown in Figure 5, the proposed method demonstrates effective navigation in indoor environments, where GNSS signals are unavailable and visibility is limited. This scenario closely reflects real-world conditions in which conventional GPS-based approaches typically fail.

The proposed method was evaluated using our own indoor campus dataset collected in a GNSS-denied environment. For comparison, the localization performance was analyzed with respect to representative visual–inertial and LiDAR–IMU methods reported in the recent literature. Since these methods were evaluated using different datasets, sensor configurations, and experimental protocols, the reported values are used only for qualitative comparison and should not be interpreted as a direct benchmark.

Therefore, a strictly fair experimental comparison with FAST-LIO2 in our indoor setup is not fully possible. Nevertheless, if we consider the reported results under ideal sensor availability as presented in [37], Our preliminary results on an approximately 400-m trajectory segment suggest that the proposed technique consistently achieves low RMSE and competitive performance compared to the evaluated baselines, as reported in the next section of this paper.

The proposed method was evaluated using our own indoor ground vehicle dataset collected in a GNSS-denied campus environment. The dataset contains approximately 400 m of robot trajectory acquired using the Delphi radar–LiDAR–camera–IMU sensor configuration. The evaluation was performed using the reference trajectory described in the previous subsection.

The proposed framework was compared with representative visual–inertial and LiDAR–IMU localization approaches, including ORB3-Stereo, VINS-Mono, VINS-Fusion, A-LOAM, FAST-LIO2, and GR-Fusion. When open-source implementations and compatible sensor configurations were available, baseline methods were evaluated on the same experimental sequences using the same evaluation protocol.

The reference trajectory was generated using a LiDAR-based localization framework, where the collected LiDAR scans were registered against a pre-built high-resolution map to obtain the reference pose sequence. The quality of the reference trajectory was verified through map-registration consistency and localization stability under the indoor experimental conditions. The Delphi radar, LiDAR, camera, and IMU measurements were synchronized using timestamp-based temporal alignment before trajectory estimation and evaluation.

Before computing localization errors, the estimated trajectories were aligned with the reference trajectory using an SE(3) transformation estimated through the least-squares method proposed by Umeyama [38]. The localization performance was evaluated using Absolute Trajectory Error (ATE) and RMSE, following the trajectory evaluation methodology introduced by Sturm et al. [39] and Kümmerle et al. [40].

**Figure 6:**
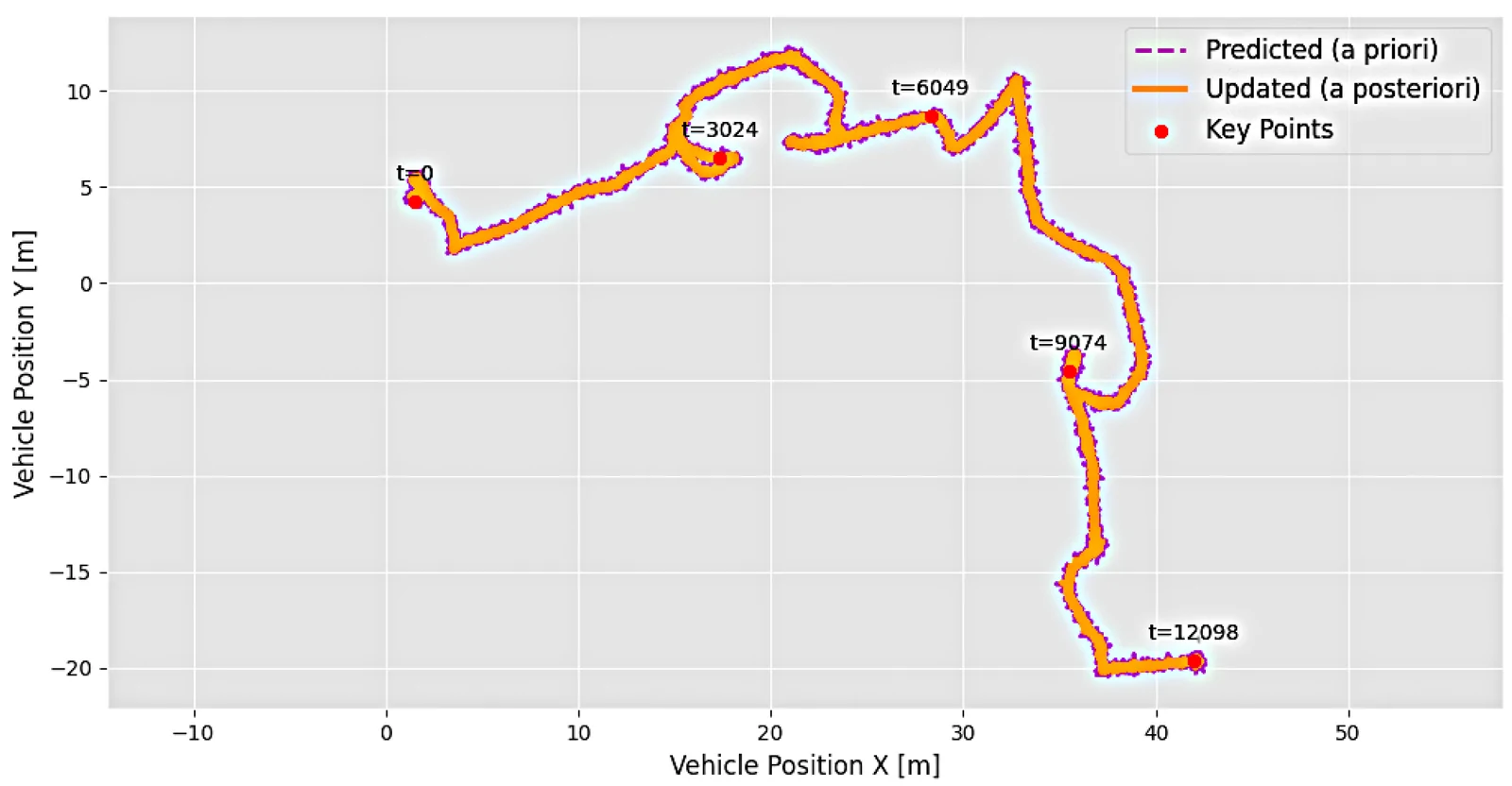
Position uncertainty reduction after multimodal fusion. The Kalman Filter is used only as a reference estimator for comparison.

The comparison between the reference trajectory and the trajectory estimated by the proposed 2M fusion framework is shown in Figure 6. The estimated trajectory remains close to the reference path, with small deviations occurring during rapid or vertical movements due to sensor noise, limited geometric constraints, and unmodeled vehicle dynamics. The axis-wise RMSE values are 0.12 m, 0.15 m, and 0.28 m along the X, Y, and Z axes, respectively, demonstrating stable localization performance across all three dimensions. The overall three-dimensional RMSE is 0.34 m. The maximum horizontal deviation was 0.45 m, while the maximum vertical deviation reached 0.62 m, confirming the robustness of the proposed framework under noisy indoor conditions.

The role of the IMU within the proposed multimodal fusion framework is illustrated in Figure 6. The IMU measurements provide high-frequency motion information that complements radar and LiDAR observations. During temporary degradation of external sensors, the learned fusion model exploits the temporal characteristics of inertial measurements to maintain trajectory continuity. Unlike conventional inertial propagation approaches, where accumulated drift is corrected explicitly through filtering, the proposed CRNN-based fusion framework learns the nonlinear relationship between inertial dynamics and spatial observations from radar and LiDAR.

**Figure 7:**
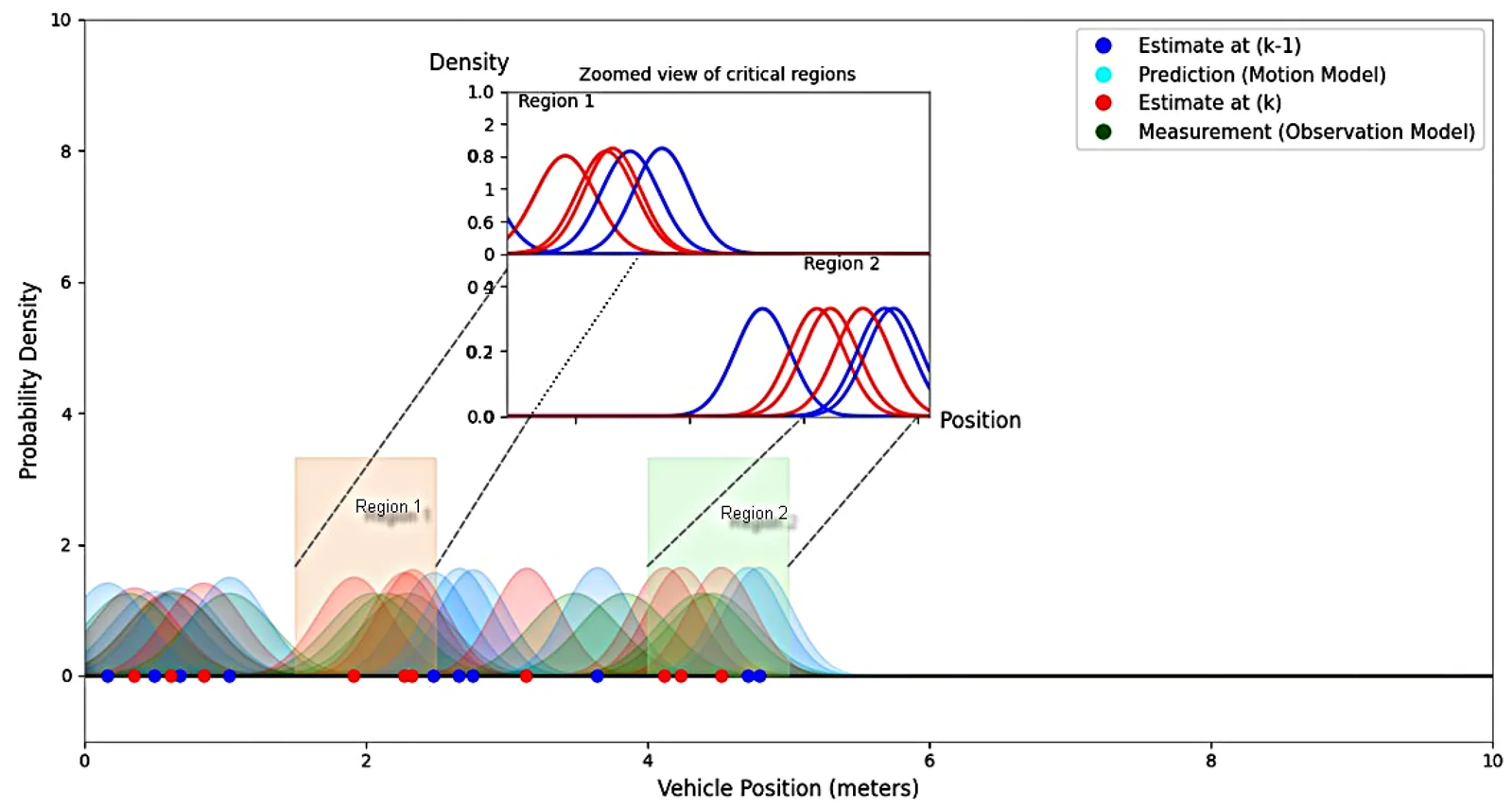
Probability Density Functions of Estimated Positions Showing Uncertainty Reduction in Critical Regions. The scaled-up view highlights the local estimation behavior, with the blue line representing the estimate at iteration (k − 1) and the red line representing the updated estimate at iteration (k). In the original figure, the sky-blue points denote the predicted positions provided by the motion model, while the green points represent the corresponding measurements obtained from the observation model.

The fusion of radar, LiDAR, and IMU measurements reduces uncertainty in the estimated trajectory. As shown in Figure 7, the probability density functions of the estimated positions become narrower after multimodal fusion, indicating improved confidence in the predicted state. This uncertainty reduction results from the complementary information provided by the heterogeneous sensors and the temporal feature extraction capability of the CRNN model rather than from a conventional Kalman correction step.

**Figure 8:**
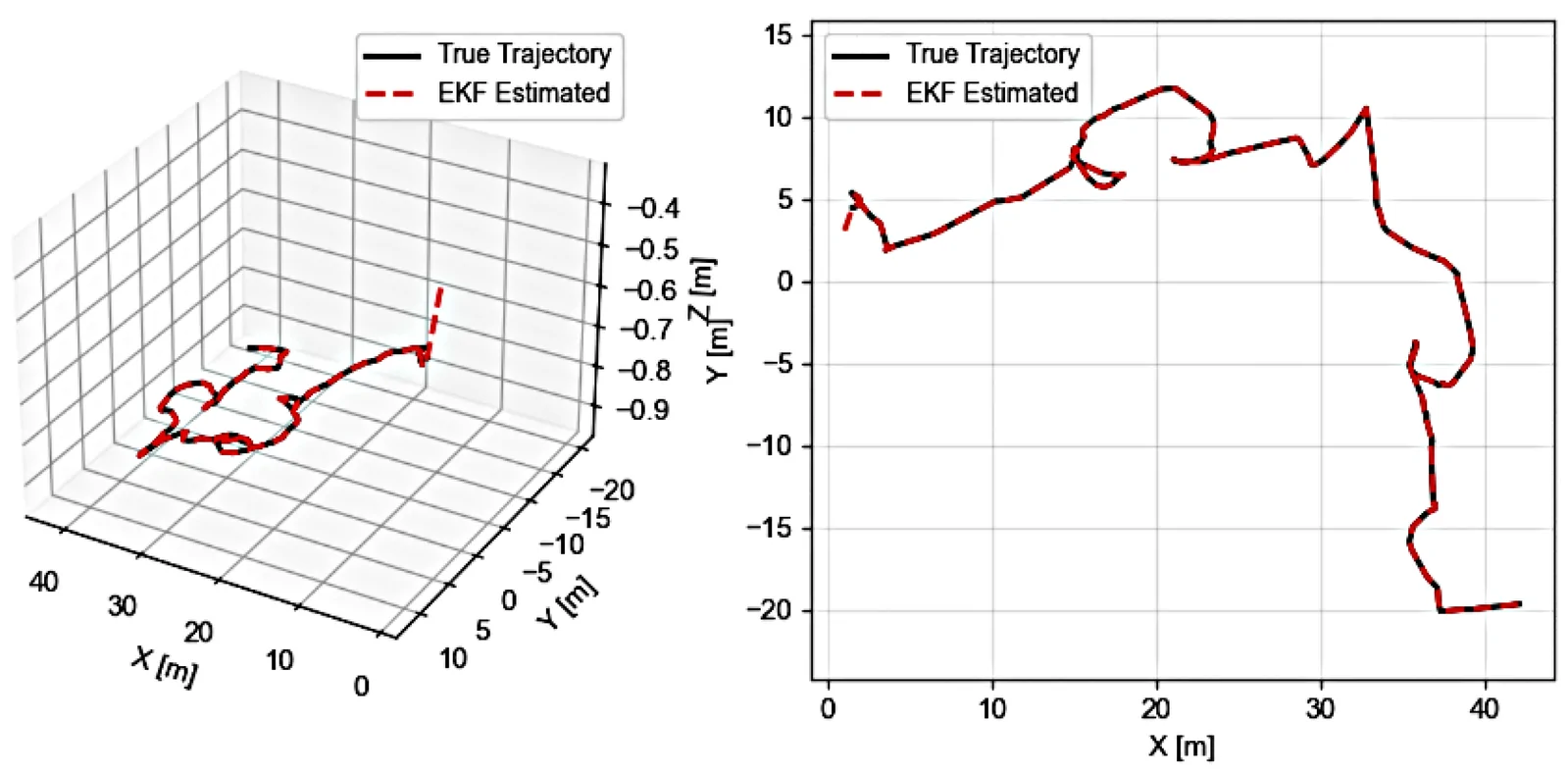
Comparison of ground-truth and proposed CRNN fusion estimated trajectories along the X, Y, and Z axes. The XY projection is shown on the (**right**), and the 3D trajectory is shown on the (**left**).

The comparison between the reference trajectory and the trajectory estimated by the proposed 2M fusion framework shown in Figure 8 demonstrates the effectiveness of the proposed approach. The estimated trajectory closely follows the reference path, particularly in the horizontal plane, while minor vertical deviations are attributed to sensor noise, environmental constraints, and unmodeled vehicle dynamics.

**Figure 9:**
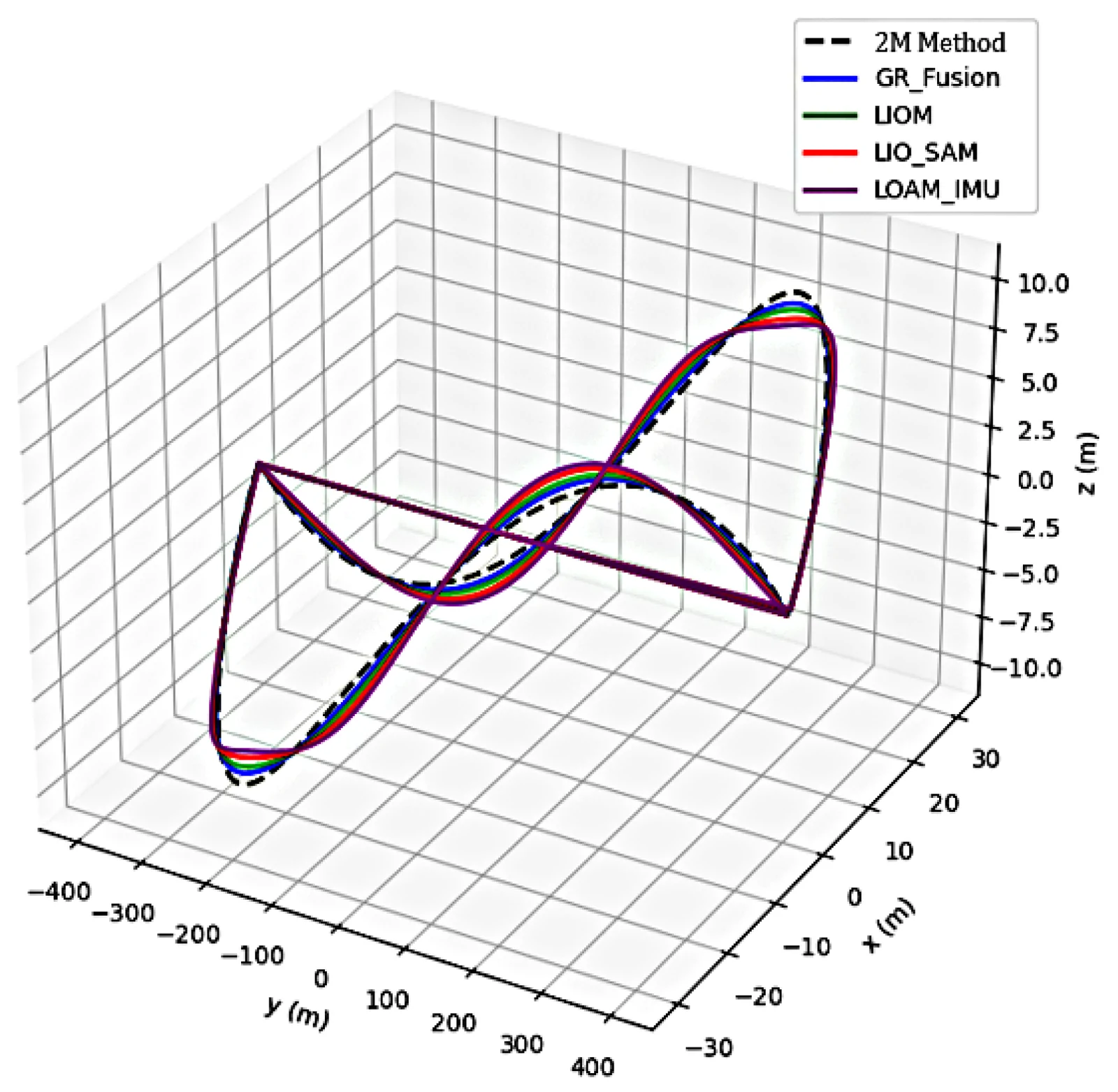
Comparison of 3D Trajectories and XY Projections of Estimated vs. True Paths.

The CRNN-based estimator attains an overall 3D localization RMSE of 0.34 m across the evaluated indoor trajectory. Figure 9 summarizes the reported RMSE values of representative LiDAR–IMU and multi-sensor localization methods from the literature. Since these methods were evaluated using different datasets, sensor configurations, and experimental protocols, the reported values are included for qualitative comparison only and should not be interpreted as a direct quantitative benchmark. Nevertheless, the proposed multimodal fusion framework demonstrates competitive localization accuracy for GNSS-denied indoor environments.

These observations indicate that the learned radar–LiDAR–IMU representation improves robustness compared with conventional filter-based and handcrafted feature-fusion approaches while maintaining competitive localization accuracy in challenging indoor environments.

**Figure 10:**
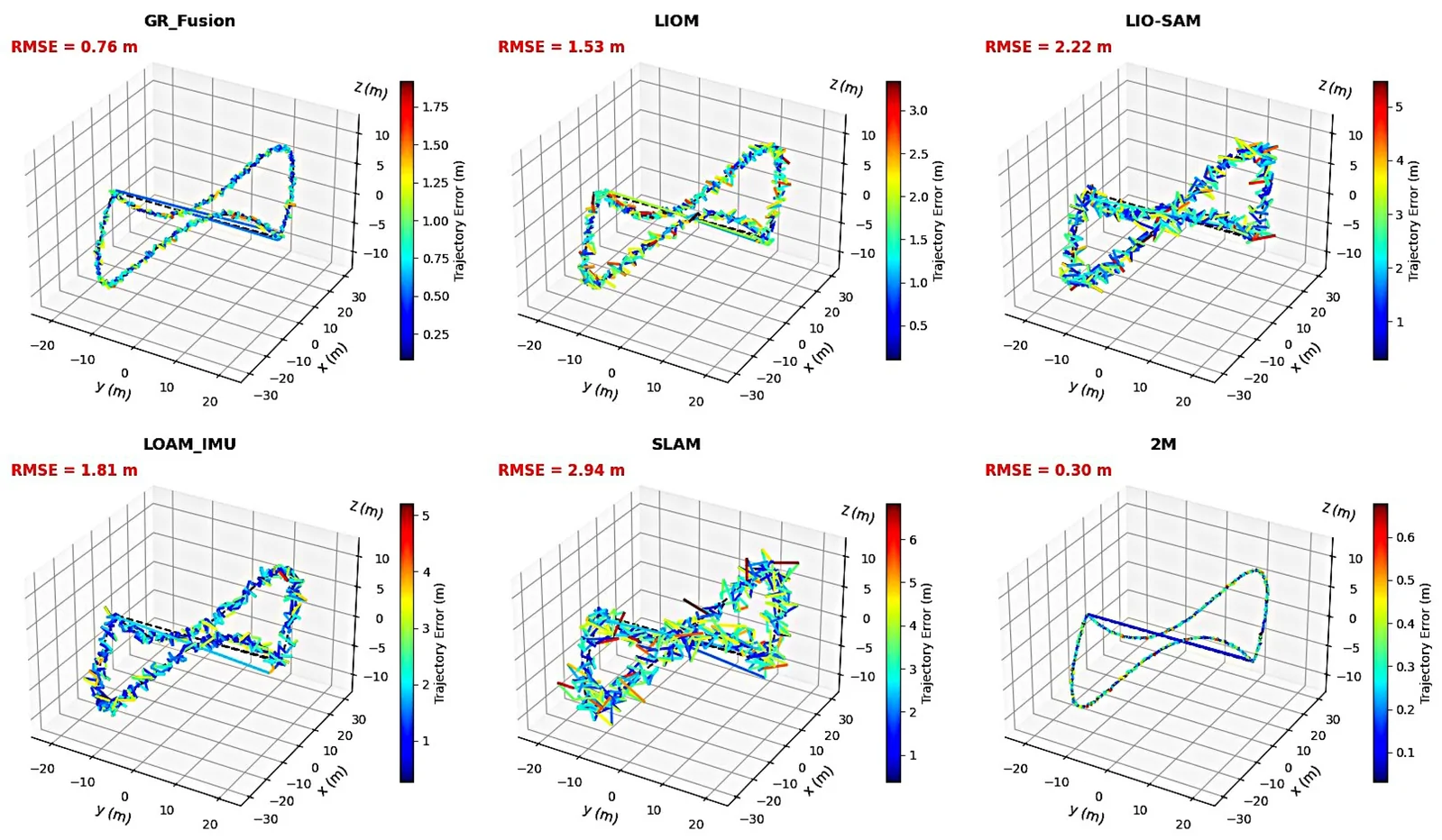
3D Results of Our Method Compared to Various Methods Based on Error Performance.

Figure 10 compares the localization performance of the proposed framework with representative methods reported in the literature. Compared with the performance reported for GR-Fusion, the proposed framework achieves a lower reported RMSE and smoother trajectory estimates on the evaluated indoor sequence. Because the experiments were conducted using different datasets, sensing configurations, and evaluation protocols, this comparison is intended to provide qualitative context rather than a direct quantitative benchmark.

The 3D error trajectories also highlight the limitations of LIO-SAM and LOAM_IMU, which exhibit pronounced Z-axis drift, visible as oscillatory deviations and spikes throughout their estimated paths. This behavior is consistent with the observations reported by Wang et al. [24]. In contrast, the proposed 2M framework maintains smoother vertical estimates with reduced Z-axis drift. This improvement is attributed to the tighter integration of radar, LiDAR, and IMU measurements within the proposed multimodal fusion architecture.

Under conditions with reduced LiDAR observability, such as underground or geometrically degraded sections, the proposed framework maintains smooth error evolution and stable localization behavior, whereas LIO-SAM and LOAM_IMU exhibit abrupt error increases. This qualitative behavior is consistent with the robustness characteristics reported for GR-Fusion, suggesting that the proposed multimodal framework effectively exploits complementary sensor information when LiDAR observations become sparse or unreliable.

The deviation statistics further support these observations. The proposed method achieved deviation percentages of 1.3% and 1.8% along the X and Y axes, respectively. For comparison, GR-Fusion reported deviation percentages of 1.5% and 1.9% on its evaluation dataset [24]. Since these values were obtained under different experimental conditions, they are presented only as qualitative reference results. Overall, the proposed framework demonstrates competitive localization performance and robustness for autonomous navigation in GNSS-denied indoor environments. Despite these results, several limitations remain. The localization accuracy is affected by significant vertical motion and highly dynamic maneuvers, where nonlinear vehicle dynamics and limited environmental constraints introduce larger estimation errors. Furthermore, the reliance on linearized Kalman filtering may reduce robustness in strongly nonlinear and non-Gaussian scenarios. To address these limitations, future work will investigate nonlinear Bayesian estimation approaches, including the Unscented Kalman Filter (UKF) proposed by Julier and Uhlmann [41] and Particle Filter (PF) methods described by Arulampalam et al. [42]. These approaches will be explored to improve uncertainty modeling, nonlinear state estimation, and robustness during aggressive motion conditions. Additional experiments on larger datasets and more diverse operating environments will also be conducted to further evaluate the scalability and generalization of the proposed framework.

Beyond improving estimation robustness, future work will extend the proposed localization framework from outdoor experimental validation toward realistic smart-city environments, where UAVs can operate as smart aerial platforms within interconnected transportation and IoT infrastructures. Before large-scale real-world deployment, simulation-based experiments will also be considered to evaluate the proposed approach under diverse traffic conditions, environmental variations, and challenging urban scenarios, using realistic autonomous-driving and robotic simulation environments such as CARLA [43]. This extension will investigate applications such as traffic monitoring, infrastructure inspection, and real-time transportation management through UAV–IoT collaboration and intelligent transportation systems [44,45]. Particular attention will be given to communication reliability, multi-sensor data fusion, data security, and privacy in complex urban environments [46]. The integration of UAV sensing, IoT, and artificial intelligence could further enable more adaptive and autonomous urban services [47]. Ultimately, this direction will assess the scalability and practical deployment of the proposed framework in dynamic, connected, and increasingly autonomous smart-city scenarios.

## 6. Conclusions

This paper presented an approach to dynamically select and combine heterogeneous sensor information within a decision-level fusion framework for robust autonomous localization in GNSS-denied environments. The framework integrates Delphi 77 GHz radar, LiDAR, and IMU measurements through a hybrid architecture that combines Kalman filtering, a Convolutional Recurrent Neural Network (CRNN), Gaussian Mixture Model (GMM)-based estimation, and a Nonlinear Autoregressive Network with eXogenous Inputs (NARX). By exploiting the complementary characteristics of these sensing modalities, the framework improves trajectory estimation reliability under sensor degradation, occlusions, and low-visibility conditions.

The decision-level fusion strategy enables the system to effectively select and combine reliable measurements from successful (i.e., on-target) trackers while reducing the impact of uncertain or degraded observations. Experimental results demonstrate that the proposed framework achieves stable and accurate three-dimensional localization on the evaluated indoor dataset, with an overall RMSE of 0.34 m. These results confirm the effectiveness of the decision-level fusion approach in maintaining accurate and robust localization by combining classical state estimation with learned multimodal representations.

Further validation in diverse real-world scenarios, including urban environments, complex road conditions, and adverse weather situations, will be essential to assess the scalability, generalization capability, and long-term robustness of the framework for future autonomous vehicle applications.

## Figures and Tables

**Table 1:** Summary of sensors and measurement specifications used in the experimental platform.

Sensor	Model	Measurement Type	Data Rate (Hz)	Field of View
LiDAR	Hesai Pandar40	3D spatial point cloud composed of range measurements, reflectivity information, and Cartesian coordinates (x,y,z) of detected points.	10–20	360° horizontal, ≈40° vertical
Radar	Delphi ESR (77 GHz FMCW)	Target range measurements (resolution: 0.1–0.5 m), radial Doppler velocity (±41m/s), azimuth angle (accuracy: 1–2°), and received signal intensity for detected targets.	20	±45° horizontal
GNSS/IMU	XW-GI7660	Inertial measurements including linear acceleration, angular velocity, position, velocity, and orientation (yaw, pitch, and roll) for vehicle pose estimation.	Up to 100	N/A

**Table 2:** Ablation study of the proposed localization framework.

Configuration	RMSE (m)	MAE (m)	Improvement (%)
Model A	0.42	0.31	0.0
Model B	0.24	0.18	42.9
Model C	0.13	0.09	69.0

**Table 3:** Prediction performance at different prediction horizons.

Prediction Horizon (s)	RMSE (m)	MAE (m)
0.2	0.11	0.08
0.5	0.15	0.11
1.0	0.21	0.16
2.0	0.29	0.22
3.0	0.38	0.27

## Data Availability

The data used in this study are not currently available through a dedicated database or platform. A resource-sharing platform is planned for the coming months in coordination with the responsible team. For further information or inquiries regarding data availability, please contact the corresponding authors.

## Abbreviations

The following abbreviations are used in this manuscript:

2M	Master Mix
AV	Autonomous Vehicle
IMU	Inertial Measurement Unit
ROS	Robot Operating System
SOTA	State-of-the-Art
ESR	Electronic Scanning Radar
FMCW	Frequency-Modulated Continuous-Wave
UWB	Ultra-Wideband
CNN	Convolutional Neural Network
LSTM	Long Short-Term Memory
GMM	Gaussian Mixture Model
DFL	Decision Fusion Level
BEV	Bird’s-Eye View
